# Evaluation of a ^68^Ga-Labeled DOTA-Tetrazine as a PET Alternative to ^111^In-SPECT Pretargeted Imaging

**DOI:** 10.3390/molecules25030463

**Published:** 2020-01-22

**Authors:** Patricia E. Edem, Jesper T. Jørgensen, Kamilla Nørregaard, Rafaella Rossin, Abdolreza Yazdani, John F. Valliant, Marc Robillard, Matthias M. Herth, Andreas Kjaer

**Affiliations:** 1 Department of Clinical Physiology, Nuclear Medicine & PET, Rigshospitalet, Blegdamsvej 9, 2100 Copenhagen, Denmark; patredem@gmail.com (P.E.E.); jespertj@sund.ku.dk (J.T.J.); kamilla.noerregaard@gmail.com (K.N.); 2 Cluster for Molecular Imaging, Department of Biomedical Sciences, University of Copenhagen, Blegdamsvej 3, 2200 Copenhagen, Denmark; 3 Department of Drug Design and Pharmacology, University of Copenhagen, Jagtvej 162, 2100 Copenhagen, Denmark; 4 Tagworks Pharmaceuticals, Geert Grooteplein Zuid 10, 6525 GA Nijmegen, The Netherlands; raffaella.rossin@tagworkspharma.com (R.R.); marc.robillard@tagworkspharma.com (M.R.); 5 Department of Chemistry and Chemical Biology, McMaster University, 1280 Main St West, Hamilton, ON L8S 4M1, Canada; ayazdani.mcmaster@gmail.com (A.Y.); valliant@mcmaster.ca (J.F.V.); 6 Pharmaceutical Chemistry and Radiopharmacy Department, School of Pharmacy, Shahid Beheshti University of Medical Sciences, PO Box 14155–6153, Tehran, Iran

**Keywords:** tetrazine ligation, PET, SPECT, gallium-68, indium-11

## Abstract

The bioorthogonal reaction between a tetrazine and strained trans-cyclooctene (TCO) has garnered success in pretargeted imaging. This reaction was first validated in nuclear imaging using an ^111^In-labeled 1,4,7,10-tetraazacyclododecane-1,4,7,10-tetraacetic acid (DOTA)-linked bispyridyl tetrazine (Tz) ([^111^In]In-DOTA-PEG_11_-Tz) and a TCO functionalized CC49 antibody. Given the initial success of this Tz, it has been paired with TCO functionalized small molecules, diabodies, and affibodies for in vivo pretargeted studies. Furthermore, the single photon emission tomography (SPECT) radionuclide, ^111^In, has been replaced with the β-emitter, ^177^Lu and α-emitter, ^212^Pb, both yielding the opportunity for targeted radiotherapy. Despite use of the ‘universal chelator’, DOTA, there is yet to be an analogue suitable for positron emission tomography (PET) using a widely available radionuclide. Here, a ^68^Ga-labeled variant ([^68^Ga]Ga-DOTA-PEG_11_-Tz) was developed and evaluated using two different in vivo pretargeting systems (Aln-TCO and TCO-CC49). Small animal imaging and ex vivo biodistribution studies were performed and revealed target specific uptake of [^68^Ga]Ga-DOTA-PEG_11_-Tz in the bone (3.7 %ID/g, knee) in mice pretreated with Aln-TCO and tumor specific uptake (5.8 %ID/g) with TCO-CC49 in mice bearing LS174 xenografts. Given the results of this study, [^68^Ga]Ga-DOTA-PEG_11_-Tz can serve as an alternative to [^111^In]In-DOTA-PEG_11_-Tz.

## 1. Introduction

Radioimmunoconjugates have gained interest as important tools in the treatment and management of cancer [1,2,3]. Their use has been applied in both radiotherapy and nuclear molecular imaging through single photon emission computed tomography (SPECT) and positron emission tomography (PET); where gamma emitting or positron emitting radionuclides are linked to a monoclonal antibody (mAb). Antibodies have the advantage of high target affinity, yet their use is hampered by poor target to non-target (T:NT) ratios due to slow target accumulation and blood clearance when compared to other targeting vectors at early time points [4]. This requires a long interval between the administration of the radioimmunoconjugate and image acquisition, thus necessitating the use of long-lived radionuclides such as indium-111 (t_1/2_ = 67.3 h) or zirconium-89 (t_1/2_ = 78.4 h) [5]. Pretargeted imaging can overcome these limitations by offering the temporal separation between administration of the mAb and the signaling agent, through a stepwise procedure.

**Figure 1:**
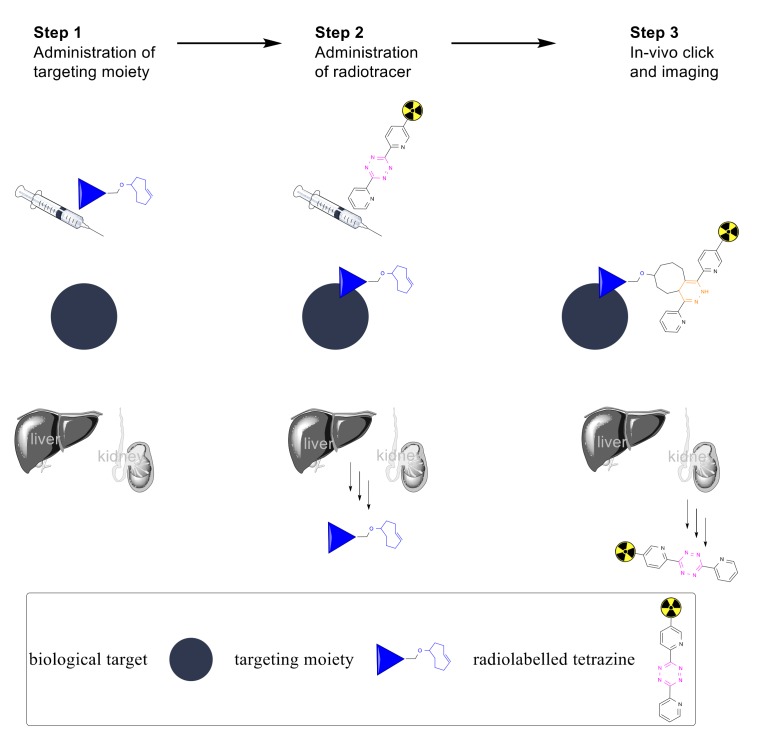
General strategy for in vivo pre-targeted imaging. In Step 1, the TCO functionalized targeted moiety is administered to the living system. In Step 2, the radiolabeled tetrazine is administered. The targeting moiety has been allowed to accumulate at the target site while any excess has cleared via the renal and/or hepatobiliary systems. In the third step in vivo imagining occurs. This is after the radioactivity has accumulated at the target site via in vivo click chemistry and any excess has cleared through the renal system.

In pretargeted imaging, a primary agent is administered and allowed to accumulate at the target site and clear from non-targeted tissues. Afterwards, a low-molecular weight radiolabeled secondary agent possessing high affinity (or reactivity) towards the primary agent is administered (Figure 1) [6]. In this way, the non-radioactive primary agent may accumulate at the target site and clear from the circulation without emitting any unnecessary radiation, while the radioactive secondary agent has the ability to clear from the circulation much faster after administration). To date, pretargeted imaging has only been validated in the clinic using the hapten-bispecific antibody, and the biotin–streptavidin interactions; however, these methods often give rise to an immunogenic response in patients [5]. The bioorthogonal chemical reaction between a tetrazine (Tz) and a trans-cyclooctene (TCO) is a chemical alternative that can mitigate these challenges. For this reason, radiolabeled tetrazines have been used extensively in pretargeted imaging with TCO functionalized mAbs (Figure 1) [6,7,8,9,10,11,12,13,14,15,16].

**Figure 2:**
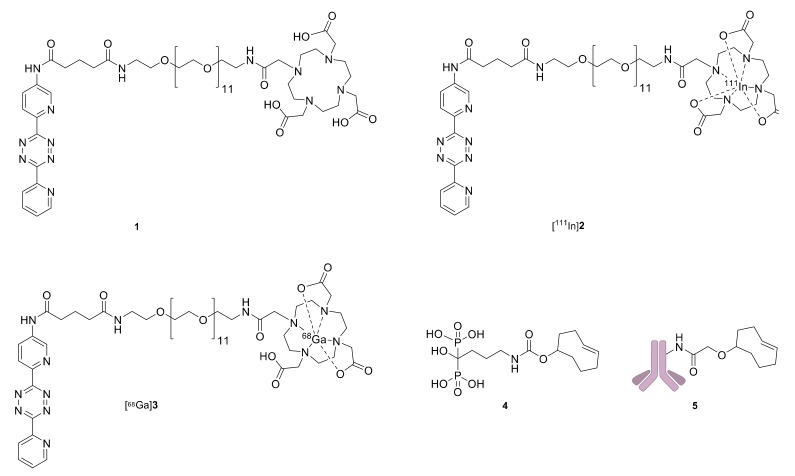
Pretargeting components used in this study: DOTA-PEG_11_-Tz (**1**); [^111^In]In-DOTA-PEG_11_-Tz (**2**); [^68^Ga]Ga-DOTA-PEG_11_-Tz (**3**); TCO functionalized alendronate (**4**); CC49-TCO (**5**).

The first radiolabeled tetrazine applied in pretargeted imaging used a 1,4,7,10-tetraazacyclododecane-1,4,7,10-tetraacetic acid (DOTA) functionalized bispyridyl tetrazine (**1**) (Figure 2) [6]. The indium labeled variant, [^111^In]**2**, was used alongside a TCO modified CC49 mAb that targets the tumor associated glycoprotein 72 (TAG-72) antigen in human colorectal cancer cells to image tumors in mice [6]. Following this seminal work, radiolabeled variants of **1** have been used in combination with different TCO functionalized primary agents such as antibodies [6,12,13,14,17,18], affibodies [19], diabodies [15], antibody drug conjugates [20] and small molecules [21,22]. These studies often incorporated long-lived radionuclides such as indium-111 [12,13,14,15,19,20], lutetium-177 (t_1/2_ = 6.6 d) [12,13,14,15,18,19,20], and lead-212 (t_1/2_ = 10.6 h) [17]. Despite DOTA forming stable complexes with a variety of metals, there are no reports of pretargeted imaging using TCO functionalized CC49 mAbs and **1** with a readily available PET based radionuclide. Given the advantage that the pretargeting strategy provides for slow clearing targeting agents, there is also an unmet need to produce an analogue of **1** radiolabeled with a short-lived radionuclide for pretargeted tumor imaging.

Here, we introduce a gallium-68 labeled variant of **1** ([^68^Ga]**3**) as an alternative to [^111^In]**2** (Figure 2). In order to evaluate [^68^Ga]**3** in mice two different primary agents were selected. We used two primary agents to demonstrate that the ability of [^68^Ga]**3** or [^111^In]**2** to bind to the respective targeting vector is independent of the targeting vector itself. TCO functionalized alendronate (**4**) was selected as the first targeting vector (Figure 2). It binds to exposed hydroxyapatite surfaces in bone [23] and has been used in pretargeting experiments to evaluate tetrazines bearing the short-lived radioisotopes, technetium-99m, (t_1/2_ = 6 h) and scandium-44 (t_1/2_ = 4 h) [21,22,23,24,25,26]. The second agent was based on the mAb CC49, which targets the TAG-72 antigen in human colorectal cancer cells. The TCO modified CC49 (**5**) has been used with [^111^In]**2** in pretargeted imaging (Figure 2) [13,14]. Initially, SPECT/CT images were obtained using [^111^In]**2** to compare the image quality at early and late time points prior to evaluating [^68^Ga]**3** at early time-points with PET/CT.

## 2. Results

### 2.1. Radiolabeling and In Vitro Stability

Radiosynthesis of [^111^In]**2** [6] was performed in high radiochemical yield (RCY) (93–100%) with high radiochemical purity (RCP > 95% by radio-HPLC) (Supplementary Figure S1). The apparent molar activity (A*_m_*) ranged from 6–13 GBq/µmol at the end of synthesis (EOS). The octanol-water partition coefficient (logD) of [^111^In]**2** was −3.659 ± 0.005. 

Radiosynthesis of [^68^Ga]**3** was accomplished in a decay corrected (d.c.) RCY of 48–78% and RCP (> 95% by radio-HPLC) (Supplementary Figure S2). The apparent A*_m_* ranged from 1–2 GBq/µmol EOS. The logD of [^68^Ga]**3** was −2.19 ± 0.03. [^68^Ga]**3**, was able to react quantitatively with **4** after 1.5 h in solution (Supplementary Figure S3A) and remained stable for at least 1.5 h at room temperature in 10% ethanol in phosphate buffered saline (PBS) (Supplementary Figure S3B).

### 2.2. Pretargeted SPECT/CT Bone Imaging

**Figure 3:**
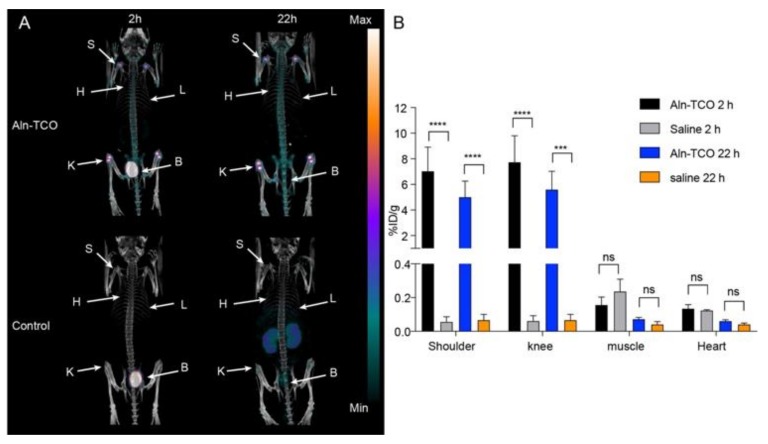
Pretargeted SPECT/CT bone imaging of [^111^In]**2** targeting TCO functionalized alendronate (**4**), which was administered 1 h prior to the administration of [^111^In]**2**. (**A**) Respective SPECT/CT images after 2 h and 22 h. (**B**) Image-derived quantitative analysis of tissue uptake. ***, *p* < 0.001; and ****, *p* < 0.0001. Each image is scaled between its minimum and maximum pixel intensity.

Prior to evaluating [^68^Ga]**3** in vivo, pretargeted bone imaging was performed using [^111^In]**2** and **4** (Figure 3). Groups of mice were pre-treated with **4** 1 h prior to administration of [^111^In]**2**. This gave a total in vivo TCO:Tz ratio of 76:1. As a control, mice that did not receive **4** were also administered [^111^In]**2**. Small animal SPECT/CT images were obtained 2 and 22 h post injection (p.i.). Radioactivity accumulation was observed in the shoulders and the knees in the mice pre-treated with **4** at both time points (see representative images in Figure 3A). Regions of interest were manually drawn on target tissues and the percent injected dose per gram of tissue (%ID/g) was extracted after dose and decay correction. The uptake in muscle was included as a measure for background accumulation. Uptake in regions of interest placed on heart tissue and used as a surrogate for blood radioactivity content. The image derived analysis showed that the mean uptake in the shoulders (7.0 ± 1.9 %ID/g 2 h p.i.; 5.0 ± 1.2 %ID/g 22 h p.i.) and knees (7.7 ± 2.1 %ID/g 2 h; 5.6 ± 1.4 %ID/g 22 h p.i.), was substantially higher at both time points than the mean uptake in the muscle (0.16 ± 0.05 %ID/g) and the heart (0.13 ± 0.02 %ID/g) (Table 1). Overall, favorable target-to-background ratios (> 40) were achieved as early as 2 h p.i. In contrast, for the non-treated animals the mean uptake values for the shoulders (0.06 ± 0.03 %ID/g) and knees (0.06 ± 0.03 %ID/g) were lower than the muscle (0.2 ± 0.1 %ID/g) and the heart (0.12 ± 0.01 %ID/g) 2 h p.i. (Supplementary Table S1). Given that the image contrast was quite good for the pre-treated mice 2 h p.i., these results indicate that **4** would be a suitable primary agent for in vivo pretargeting with the short-lived radionuclide, gallium-68.

### 2.3. Pretargeted PET/CT Bone Imaging

**Figure 4:**
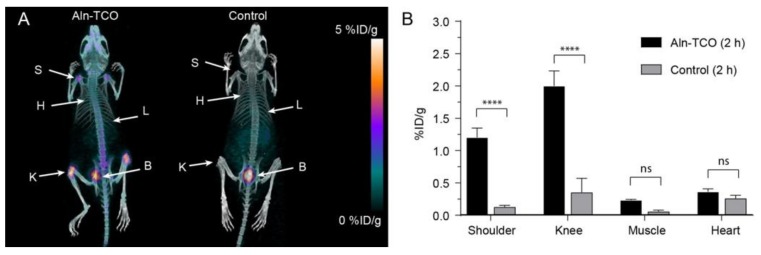
Pretargeted PET/CT bone imaging of [^68^Ga]**3** targeting TCO functionalized alendronate (**4**), which was administered 1 h prior to the administration of [^68^Ga]**3**. (**A**) Respective PET/CT images after 2 h. (**B**) Image-derived quantitative analysis of tissue uptake. **** *p* < 0.0001.

In a similar fashion [^68^Ga]**3** was evaluated in pretargeted PET imaging using **4** as the primary agent. Mice were injected with **4** 1 h prior to administration of [^68^Ga]**3**, giving a total in vivo TCO:Tz ratio of 25:1. Mice that that did not receive **4** were also given [^68^Ga]**3** as a control. PET/CT images were obtained 2 h p.i. In the treatment group, the shoulders and knees were clearly visualized (Figure 4). The uptake was quantified and the mean uptake values in the shoulders and knees of the pre-treated mice were found to be 1.20 ± 0.01 %ID/g and 2.0 ± 0.2 %ID/g, respectively (Figure 4B; Table S1). High radioactivity uptake was also observed in the bladder, similar to what was observed in the SPECT/CT images 2 h after administering [^111^In]**2**. There was no substantial uptake in the bone when there was no pre-treatment with **4** (≥ 0.4 %ID/g) (Supplementary Table S1). Although the target areas were clearly visualized, the PET derived target-to-background ratios were lower (< 8.6) than those obtained from the analogous SPECT experiment.

Following the last scan shoulder, knee, and muscle tissue was collected and counted for radioactivity. The mean uptake values were 2.3 ± 0.5 %ID/g for the shoulders and 3.7 ± 0.6 %ID/g for the knees. The uptake in the muscle was quite low (0.10 ± 0.04 %ID/g) (Table 1). Overall, pretargeting using **4** provided a fairly good image contrast with low background uptake using both [^111^In]**2** and [^68^Ga]**3**.

### 2.4. Pretargeted SPECT/CT Tumor Imaging

**Figure 5:**
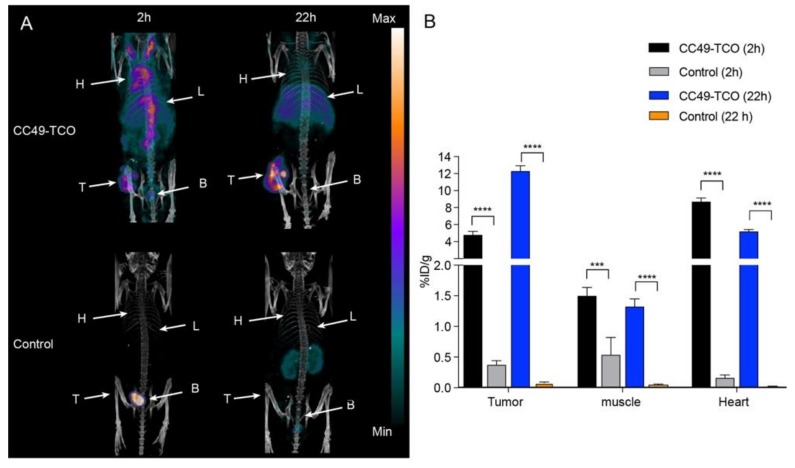
Pretargeted SPECT/CT imaging of [^111^In]**2** targeting CC49-TCO (**5**), which was administered 24 h prior to the administration of [^111^In]**2**. (**A**) Respective SPECT/CT images after 2 h and 22 h. (**B**) Image-derived quantitative analysis of tissue uptake. *** *p* < 0.001 and **** *p* < 0.0001. Each image is scaled between its minimum and maximum pixel intensity.

Given that we were able to obtain high contrast images with [^68^Ga]**3** using **4** as the primary agent, we were encouraged to evaluate this tetrazine with a slow clearing primary agent. Prior to evaluating [^68^Ga]**3**, [^111^In]**2** was tested as a positive control alongside **5**. Mice bearing LS174T tumor xenografts in the left flank were injected intravenously with **5** one day prior to administration of [^111^In]**2**. The total in vivo TCO:Tz ratio was 1:2. As a control, mice bearing the LS174T tumor xenografts, without any pre-treatment, were also injected with [^111^In]**2**. SPECT/CT images were obtained 2 and 22 h p.i. Representative SPECT/CT images and the quantified mean uptake values are shown in Figure 5. The tumor was clearly visualized in mice pre-treated with **5** (Figure 5A) at both time points, although the background was quite high at 2 h p.i. The mean heart uptake (8.7 ± 0.4 %ID/g) was used as a surrogate for activity in the blood pool and was found to be almost twice as high as the mean uptake determined for the tumor (4.8 ± 0.4 %ID/g) (Figure 5B; Table 2). After 22 h, the mean uptake was reduced in all tissues except for the tumors, where the uptake value increased to 12.3 ± 0.6 %ID/g. The tumor-to-background ratios did increase overtime with a maximal value of 9.2 for the tumor-to-muscle ratio 22 h p.i. (Table 2). The high background activity at 2 h p.i. combined with the increased tumor uptake at 22 h p.i. indicates a portion of **5** remained in the circulation and then reacted with [^111^In]**2** in the blood following administration. In contrast, there was no substantial uptake in the tumor, muscle, or heart in the control animals (< 0.6 %ID/g) (Supplementary Table S2).

### 2.5. Pretargeted PET/CT Tumor Imaging

**Figure 6:**
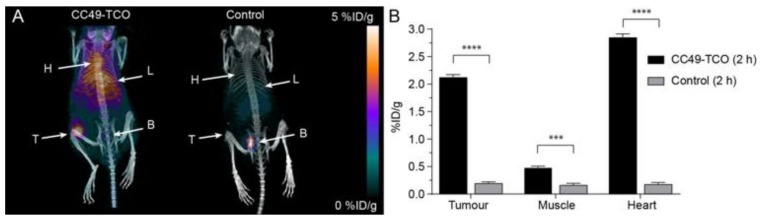
Pretargeted PET/CT imaging of [^68^Ga]**3** targeting CC49-TCO (**5**), which was administered 24 h prior to the administration of [^68^Ga]**3.** (**A**) Respective PET/CT images after 2 h. (**B**) Image-derived quantitative analysis of tissue uptake. *** *p* < 0.001 and **** *p* < 0.0001.

Following SPECT/CT imaging, pretargeted tumor imaging was performed using **5** as the primary agent and [^68^Ga]**3** as the secondary agent. Mice bearing LS174T tumor xenografts in the left flank were administered **5** 24 h prior to administration of [^68^Ga]**3**, giving a total in vivo TCO:Tz ratio of 1:2. Control mice bearing tumor xenografts without pre-treatment were also given [^68^Ga]**3**. PET/CT images were obtained 2 h p.i. Similar to the indium-111 studies, the tumor was clearly visualized; however, high background activity was also observed (Figure 6A). Nevertheless, the mean tumor uptake was higher in the pre-treated mice (2.1 ± 0.1 %ID/g) when compared to the tumor uptake in the control mice (0.19 ± 0.03 %ID/g) (Figure 6B; Table S2). Again, a considerable amount of activity was observed in the heart (2.9 ± 0.1 %ID/g) of the pre-treated animals compared to the controls (0.18 ± 0.03 %ID/g) indicating that the tetrazine ligation occurs with residual **5** circulating in the blood (Table 2: Supplementary Table S2). 

Following the last scan, tumor, muscle, and blood samples were collected and radioactivity was measured. The mean uptake value for the tumor was 5.8 ± 0.3 %ID/g, the tumor-to-muscle ratio was 9.8 and the tumor-to-blood ratio was 1.1 (Table 2).

## 3. Discussion

We report the radiosynthesis of a ^68^Ga-labeled DOTA tetrazine ([^68^Ga]**3**) as a novel PET analogue to [^111^In]**2**. The radiosynthesis of [^68^Ga]**3** was performed using methods similar to previous reports [27], however, modifications were put in place so that the reaction conditions were similar to that of the indium-111 labeling. The reaction was fast (10 min) and efficient, resulting in total incorporation of the gallium-68 available in solution. The less than quantitative RCY can be attributed to nonspecific binding of radioactive components to the reaction vessel and SPE cartridge during reformulation.

Pretargeting using a radiolabeled tetrazine and TCO functionalized mAb was established as an efficient method for imaging tumors in 2010 [6]. Since then, various pretargeting experiments involving the TCO modified CC49 antibodies have been optimized resulting in improved image quality for [^111^In]**2** [11,12,13]. One strategy that showed to significantly improve the image contrast involved the use of a tetrazine functionalized clearing agent to remove any residual mAb from the circulation. It has also been reported that increasing the amount of non-labeled tetrazine can reduce the blood capture of the radiolabeled tetrazine by any residual circulating TCO-functionalized antibodies [28]. Apart from this, great effort has been put into the development of additional radiolabeled tetrazines as potential pretargeting imaging agents. However, many reports of these new radiolabeled tetrazines lack in vivo evaluation of their use as pretargeted imaging agents, likely due to the lack of alternative pretargeting screening models to mAbs [29,30,31,32,33,34]. Although TCO-CC49 mAb serves as a positive control for pretargeting experiments because of its extensive optimization and evaluation, antibodies as a screening model is challenged by the need of meticulous pretargeting procedures, disease models to test them, and the fact that they and their TCO modification can be expensive to produce. One alternative to TCO-modified antibodies is TCO-linked bisphosphonates, alendronate (Aln), that can be used for pretargeted imaging of shoulder and knee joints in healthy mice and offers a much simpler experimental procedure for screening new tetrazines [21,35,36].

Hence, in this study, TCO-modified CC49 mAb and Aln were selected to evaluate the utility of [^68^Ga]**3** as a PET isotope labeled variant of [^111^In]**2**. We found that healthy mice pre-treated with bisphosphonates gave reproducible ^111^In-SPECT images with good contrast between the joints and background as early as 2 h p.i., which is a suitable time-point for ^68^Ga-PET. Analysis showed that the uptake in the shoulder and the knees was nearly identical at 2 and 22 h p.i., respectively. This is expected as the IEDDA reaction is fast and irreversible, yielding a stable ligation product and because the unreacted TCO and Tz moieties are rapidly cleared from the system [5,21]. Using [^68^Ga]**3** and 2 h p.i. PET imaging, we again found that mice pre-treated with **4** had a significantly higher uptake in shoulder and knees compared to both control tissues (muscle and heart) and mice with no pre-treatment. Interestingly, the target uptake was higher using the [^111^In]**2** compared to [^68^Ga]**3**. However, a higher TCO:Tz ratio was used for the [^111^In]**2** study potentially resulting in increased accumulation as a result of higher number of TCO in target tissue. Also, indium can form 7 or 8 coordinate DOTA complexes, whereas gallium forms 6 coordinate species [37,38,39]. This chemical difference can result in different biodistribution patterns for ^68^Ga and ^111^In-labeled radiotracers [40,41]. Furthermore, since Ga-DOTA complexes are less stable than In-DOTA complexes, there is a greater risk for in vivo demetallation [40,41]. All these factors may contribute to the fact that lower accumulation was observed with [^68^Ga]**3** compared to [^111^In]**2**. Regardless, the image contrast of [^68^Ga]**3** was quite good 2 h and 22 h p.i. 

Although previous optimization studies with **5** showed improved contrast with the use of a clearing agent, we opted to forgo this to simplify our approach. Instead, we employed a TCO:Tz ratio of 1:2 compared to a 1:1 ratio that was used in presence of a clearing agent. Mice bearing LS174T tumor xenografts were pre-treated with **5** 24 h prior to receiving [^111^In]**2**, and SPECT/CT scanned 2 and 22 h p.i. Tumors were clearly visualized at both time points, however, the image contrast significantly improved at 22 h p.i. Image derived uptake analysis also showed that the tumor uptake increased more than two-fold from 2 h to 22 h p.i., whereas the activity was reduced in the muscle and heart. In a similar way, PET/CT imaging with [^68^Ga]**3** 2 h p.i. provided a fairly good tumor uptake, however, the image contrast was impaired by the very high background. In contrast, tumor-bearing mice that did not receive any pre-treatment, showed negligible uptake in all tissues with both [^68^Ga]**3** and [^111^In]**2**. Given the higher blood activity levels in the pretargeted experiments in comparison to the controls (Figure 5 and Figure 6), it appears that the radiolabeled tetrazines are captured by residual antibodies in the blood pool. Following IEDDA reaction the ligation product can slowly accumulate in the tumors, increasing the uptake over time. 

For [^68^Ga]**3**, there was some deviation between the uptake values from the ex vivo and the image derived analysis, e.g., most notably we found a mean tumor uptake of 2.1 %ID/g using PET and 5.8 %ID/g using a gamma counter. The tumors used in these studies were somewhat small (≈125 mm^3^) and therefor these deviations probably relate to the positron range of ^68^Ga (R_mean_: 2.9 mm; R_max_: 8.2 mm (in water) [42]. For ^68^Ga this is a particular a problem in small animal-imaging with high spatial resolution, and where the target tissue often is smaller or on the same scale as the positron range. This has also previously been reported by others [42]. However, in clinical settings this is less of a problem and ^68^Ga, as a PET isotope, has the advantage of being produced from a relatively inexpensive and easily accessible generator system.

## 4. Material and Methods

### 4.1. General

All reagents and solvents were purchased from Sigma-Aldrich, unless otherwise, stated and used without further purification. [^111^In]InCl_3_ (non-carrier added) was purchased from Lægemiddel Styrelsen. [^68^Ga]GaCl_3_ was produced from ^68^Ga/^68^Ge generator (Eckert & Ziegler) and was eluted with 0.1 M HCl in fractions (1 mL). As these are both radioactive substance proper facilities, licenses, and procedures must be put in place prior to use. Water was distilled and deionized using a Milli-Q water filtration system (Millipore). Buffers used in the ^111^In-labelings were treated with Chelex-100 resin overnight, filtered (0.22 µm) and stored at 4 °C. Analytical radioactive thin-layer chromatography (radio-TLC) was performed using silica gel plates (Sigma-Aldrich) or C18 silica gel (Merk) with fluorescent indicator UV254 and visualized using a TLC scanner. Analytical high-performance liquid chromatography (HPLC) was performed using a Thermo Scientific system equipped with a photodiode array (PDA) detector and a Gabi radioactivity detector (Raytest). Compounds were eluted with a Chromalith Performance RP-18 endcapped 100-4.6 HPLC column using the following elution conditions: Solvent A = water with 0.1% trifluoroactic acid (TFA), solvent B = MeCN with 0.1% trifluoracetic acid (TFA). Gradient: 5% B to 95% B, 0–10 min. The flow rate was set at 2.5 mL/min. UV monitoring occurred at 254 nm. 

### 4.2. Statistics 

Statistical analysis was performed in GraphPad Prism 8. Unless stated otherwise all values are mean ± s.e.m. The tissue uptake was compared with either unpaired t-test or one-way ANOVA with multiple comparion and Sidak’s post hoc test. All statistical results were considered significant when *p*-value < 0.05.

### 4.3. Synthesis of Pretargeting Components

Aln-TCO (**4**) [21], CC49−TCO (**5**) [13,14], and the DOTA−tetrazine (**1**) [6] were prepared as previously described.

### 4.4. Radiochemistry—Labeling of ***1*** with Indium-111 

[^111^In]Indium-2,2′,2″-(10-(2,40,44-trioxo-44-((6-(6-(pyridin-2-yl)-1,2,4,5-tetrazx-yl)pyridx-yl)amino)-6,9,12,15,18,21,24,27,30,33,36-undecaoxa-3,39-diazatetratetracontyl)-1,4,7,10-tetraazacyclododecane-1,4,7-triyl)triacetic acetate ([^111^In]**1**). [^111^In]Indium chloride (480–615 MBq) in 0.1 M HCl (1 mL) was added to a vial containing **1** (50–100 µg, 0.04–0.08 µmol) in 0.2 M ammonium acetate pH 7 (25–50 µL) and heated at 60 °C. After 10 min diethylenetriamine-pentaacetic acid (DTPA) (19 µg, 0.05 µmol) in PBS (5 µL) and gentisic acid (588 µg, 3.8 µmol) in saline (29.4 µL) was added and the solution was heated for an additional 5 min. RCY ndc = 93%-quantitative (n = 10); Radiochemical purity (RCP) > 95%; A_m_: 6–16 GBq/µmol; HPLC (method A) R_t_ = 3.26 min. Radio-TLC (200 mM EDTA in saline) R_f_ = 0.

### 4.5. Labeling of ***3*** with Galium-68 

[^68^Ga]Gallium-2,2′,2″-(10-(2,40,44-trioxo-44-((6-(6-(pyridin-2-yl)-1,2,4,5-tetrazx-yl)pyridx-yl)amino)-6,9,12,15,18,21,24,27,30,33,36-undecaoxa-3,39-diazatetratetracontyl)-1,4,7,10-tetraazacyclododecane-1,4,7-triyl)triacetic acetate ([^68^Ga]**3**). [^68^Ga]Gallium chloride (164–278 MBq) in 0.1 M HCl (1–2 mL) was eluted directly to a reaction vial containing **1** (50–100 µg, 0.04–0.08 µmol) in 0.2 M ammonium acetate pH 7 (25–50 µL), 70% ethanol (400 µL) and 0.2 M ammonium acetate pH 6 (1 mL). The reaction was heated at 90 °C for 10 min. After cooling to room temperature, the reaction was loaded on a solid phase extraction (SPE) cartridge. The SPE cartridge was pre-rinsed 70% ethanol (5 mL) and water (10 mL). Unreacted [^68^Ga]GaCl_3_ was eluted with water (10 mL) and the product was eluted with absolute ethanol (1 mL). RCY dc = 48–78% (n = 4); RCP > 90%; molar activity 2–6 GBq/μmol; HPLC R_t_ = 3.34 min; Radio-TLC (0.1 M citrate buffer, pH 4) R_f_ = 0.

### 4.6. Determination of Log D (pH 7.4)

The octanol–water partition coefficient (Log D) was measured in a similar manner previously described [43].

### 4.7. In Vitro Stability

Solutions of [^111^In]**2** were prepared in 0.2 M ammonium acetate at pH 5.5 and incubated at room temperature. Aliquots (1 µL) were taken at 5, 30, 45, 60, and 90 min. After 90 min an additional aliquot (1 µL) was added to **4** (10 µg, 0.02 µmol) in saline (10 µL). After 5 min samples were analyzed using RP radio-TLC. RP radioTLC (50% MeCN/H_2_O) R_f_([^111^In]**2**) = 0.9; R_f_([^111^In]**2** + **4**) = 0.0.

Solutions of [^68^Ga]**3** were formulated in 10% EtOH/PBS and incubated at room temperature. Studies were performed as described for [^111^In]**2**. RP radioTLC (50% MeCN/H_2_O) R_f_([^68^Ga]**3**) = 0.2; R_f_([^68^Ga]**3** + **4**) = 0.0.

### 4.8. In Vivo Studies

All animal experiments were approved by the Danish Animal Welfare Council, Ministry of Justice and performed in accordance with the approved guidelines. Five-week-old female BALB/c nu/nu mice and female BALB/c mice were obtained from Charles River Laboratories and allowed to acclimatize for one week in the animal facility. At all times, they had access to water and feed ad libitum. LS174T human colon cancer cell line (ATCC) was cultured in minimum essential medium (MEM) supplemented with 10% fetal bovine serum (FBS), 1% L-Glutamine, 1% Sodium pyruvate, 1% non-essential amino acids, and 1% penicillin-streptomycin (Pen Strep) at 37 °C and 5% CO_2_. At a confluence of 70–90%, cells were harvested by trypsination and subcutaneous tumors were established in the left flank of the 6 weeks old BALB/c nu/nu mice by inoculation of ~5 × 10^6^ LS174T cells resuspended in sterile PBS (100 μL). Tumors were allowed to grow for 7–10 days before use. All animals were euthanized after their last scan. To determine tumor size, dimensions were measured using a caliper and the volume calculated as: volume = 1/2(length × width^2^).

SPECT/CT imaging was conducted using a small animal dedicated nanoSPECT/CT system (Mediso). CT images were acquired using the semicircular multi field of view (FOV) method in Nucline Software and using the settings: 720 projections, 35 kVp, 980 µA, 450 ms, 1:4 binning. SPECT images were acquired at 245.4 keV (20% full width) as primary photopeak and 171.3.4 keV (20% full width) as secondary photopeak. Each projection was obtained with 50,000–100,000 counts, except for the control experiments with only 15,000 counts. In the Nucline Software, CT images were reconstructed using small voxel size, thin slice thickness, and cosine filter and SPECT images were reconstructed using Tera-Tomo^TM^ 3D SPECT reconstruction software. PET/CT imaging was conducted on a small animal dedicated Inveon PET/CT system (Siemens). Static PET images were acquired with an energy window of 350–650 KeV and a time resolution of 6 ns. CT scans were acquired using 360 projections, 65 kV, 500 μA, and 400 ms. PET images were reconstructed using a three-dimensional maximum a posteriori algorithm with CT-based attenuation correction. SPECT/PET and CT images were fused and co-registered and analyzed using either VivoQuant software (SPECT; Invicro) or Inveon software (PET; Siemens). The mean percentage of injected dose per grams of tissue (%ID/g) in the tissue volume was extracted by manually drawing regions of interest on the entire tissue and correct for decay and injected amount of radioactivity. During all scans, animals were kept anesthetized by breathing 3–5% sevoflurane mixed with 35% O_2_ in N_2_ and their body temperature maintained either by a temperature-controlled air flow or by being placed on a heating pad.

Regions of interest were manually drawn on target tissues and the percent injected dose per gram of tissue (%ID/g) was extracted after dose and decay correction.

#### 4.8.1. Evaluation of [^111^In]**2** in Mice Pretreated with **5**

Four female nude BALB/c mice bearing subcutaneous LS174T tumor xenografts (mean size of 111.8 ± 29.7 mm^3^) were administered i.v. with **5** (100 µg) in PBS (50 µL). The control group for **5** was nude BALB/c mice bearing LS174T subcutaneous tumor xenografts (mean size of 136.7 ± 13.1 mm^3^). After 24 h, [^111^In]**2** (mean 59 MBq, 12 µg, 9.7 nmol) in 0.2 M ammonium acetate pH 5 (50 µL) was administered i.v. SPECT/CT images were obtained 2 and 22 h p.i.

#### 4.8.2. Evaluation of [^111^In]**2** in Mice Pretreated with **4**

Three female BALB/c mice were administered i.v. with **4** (100 µg, 0.25 µmol) in saline (100 µL). As a control group, we used three mice without pre-treatment. After 1 h [^111^In]**2** (mean 43 MBq, 4.2 µg, 3.3 nmol) in 0.2 M ammonium acetate pH 5 (100 µL) was administered i.v. SPECT/CT images were obtained 2 and 22 h p.i. 

#### 4.8.3. Evaluation of [^68^Ga]**3** in Mice Bearing LS174T Tumor Xenografts 

Four female nude BALB/c mice bearing subcutaneous LS174T tumor xenografts were administered i.v. with **5** (100 µg) in PBS (50 µL); four mice without pre-treatment were included as controls (mean size of 126.2 ± 26.0 mm^3^). After 24 h [^68^Ga]**3** (mean 6 MBq, 10–13 µg, 8–10 nmol) in < 10% EtOH/PBS (100µL) was administered i.v. PET/CT images were obtained 2 h p.i. Following last scan, the mice were euthanized and the tumor, blood, and muscle tissue were collected and weighed. Sample radioactivity was measured using a gamma counter and the %ID/g values were calculated after correcting for decay and injected amount of radioactivity.

#### 4.8.4. Evaluation of [^68^Ga]**3** in Mice Pre-Treated with **4**

Female BALB/c mice were administered i.v. with **4** (100 µg, 0.25 µmol) in saline (100 µL) (n = 4). Four mice without pre-treatment were included as controls. After 1 h [^68^Ga]**3** (mean 11 MBq, 8–13 µg, 6.4–10 nmol) in < 10% EtOH/PBS (100 µL) was administered i.v. PET/CT images were obtained 2 p.i. Following the scan, the mice were euthanized, and the knee, shoulder, and muscle tissue were collected and weighed. Sample radioactivity was measured using a gamma counter and the %ID/g values were calculated after correcting for decay and injected amount of radioactivity.

## 5. Conclusions

Within this study, we demonstrated that [^68^Ga]**3** can serve as a PET alternative for pretargeted imaging, even though it displayed lower accumulation values than the ‘Gold-standard’ for pretargeted imaging, [^111^In]**2.**

## Figures and Tables

**Table 1:** Uptake values in selected tissues from SPECT/CT scans 2 h and 22 h after injection of [^111^In]**2** in healthy BALB/c mice (*n* = 3) pretreated with **4** and from PET/CT scans and ex vivo (gammacounter) 2 h after injection of [^68^Ga]**3** in healthy BALB/c mice (*n* = 4) pretreated with **4**. Data is given as mean ± standard error of mean (SEM). * Image-derived uptake in heart from SPECT and PET images used as a surrogate for the blood radioactivity content. ** Not measured ex vivo.

	Pretargeted [^111^In]2 (%ID/g)		Pretargeted [^68^Ga]3 (%ID/g)	
	SPECT 2 h	SPECT 22 h	PET 2 h	Ex Vivo 2 h
Shoulder	7.0 ± 1.9	5.0 ± 1.2	1.20 ± 0.01	2.3 ± 0.5
Knee	7.7 ± 2.1	5.6 ± 1.4	2.0 ± 0.2	3.7 ± 0.6
Muscle	0.16 ± 0.05	0.07 ± 0.01	0.23 ± 0.01	0.10 ± 0.04
Heart *	0.13 ± 0.02	0.06 ± 0.01	0.36 ± 0.05	-**

**Table 2:** Uptake values, tumor-to-muscle (T/M) ratios and tumor-to-blood (T/B) ratios from [^111^In]**2** SPECT/CT (*n* = 3) and [^68^Ga]**3** PET/CT and ex vivo (gammacounter) (*n* = 4) in selected tissues in nude BALB/c mice bearing subcutaneous LS174T tumor xenografts pretreated with **5**. Data is given as mean ± standard error of mean (SEM). * Image-derived uptake in heart from SPECT and PET images used as a surrogate for the blood radioactivity content. ** Radioactivity content in blood measured using gamma counter. *** Tumor-to-blood ratio based on image-derived radioactivity content found in regions of interest created on tumor and heart tissue.

	Pretargeted [^111^In]2 (%ID/g)		Pretargeted [^68^Ga]3	
	SPECT 2 h	SPECT 22 h	PET 2 h	Ex Vivo 2 h
Tumor	4.8 ± 0.4	12.3 ± 0.6	2.1 ± 0.1	5.8 ± 0.3
Heart (blood)	8.7 ± 0.4 *	5.2 ± 0.2 *	2.9 ± 0.1 *	5.5 ± 0.1 **
Muscle	1.5 ± 0.1	1.3 ± 0.1	0.48 ± 0.02	0.6 ± 0.1
T/M ratio	3.2	9.2	4.4	9.8
T/B ratio	0.6 ***	2.4 ***	0.8 ***	1.1

## Supplementary Materials

The following are available online. Analytical HPLC data of [111In]2 and [68Ga]3 (Figures S1 and S2), RadioTLC data of [68Ga]3 (Figure S3), additional PET biodistribution data (Table S1) and SPECT biodistribution data (Table S2) are reported.
