# Supplementary material for: Evaluation of a 68Ga-Labeled DOTA-Tetrazine as a PET Alternative to 111In-SPECT Pretargeted Imaging
Source: Molecules. 2020 Jan 22;25(3):463. doi: 10.3390/molecules25030463 (PMC7036891; doi:10.3390/molecules25030463)
Supplement: Supplementary file 1 [file molecules-25-00463-s001.pdf]

molecules

Evaluation of a ^{68}Ga-labelled DOTA-tetrazine as a PET alternative to ^{111}In-SPECT pretargeted imaging

Patricia E. Edem [1,2,3,†], Jesper T. Jørgensen [1,2,†], Kamilla Nørregaard [1,2], Rafaella Rossin [4], Abdolreza Yazdani [5,6], John F. Valliant [5], Marc Robillard [4], Matthias M. Herth [1,3,*], and Andreas Kjaer [1,2,*]

[1] Department of Clinical Physiology, Nuclear Medicine & PET, Rigshospitalet, Blegdamsvej 9, 2100 Copenhagen, Denmark; patredem@gmail.com (P.E.E.); jespertj@sund.ku.dk (J.T.J.); kamilla.noerregaard@gmail.com (K.N.); matthias.herth@sund.ku.dk (M.M.H.); akjaer@sund.ku.dk (A.K.)
[2] Cluster for Molecular Imaging, Department of Biomedical Sciences, University of Copenhagen, Blegdamsvej 3, 2200 Copenhagen, Denmark
[3] Department of Drug Design and Pharmacology, University of Copenhagen, Jagtvej 162, 2100 Copenhagen, Denmark
[4] Tagworks Pharmaceuticals, Geert Grooteplein Zuid 10, 6525 GA Nijmegen, The Netherlands; raffaella.rossin@tagworkspharma.com (R.R.); marc.robillard@tagworkspharma.com (M.R.)
[5] Department of Chemistry and Chemical Biology, McMaster University, 1280 Main St West, Hamilton, ON L8S 4M1, Canada; ayazdani.mcmaster@gmail.com (A.Y.); valliant@mcmaster.ca (J.F.V.)
[6] Pharmaceutical Chemistry and Radiopharmacy Department, School of Pharmacy, Shahid Beheshti University of Medical Sciences, PO Box 14155–6153, Tehran, Iran
* Correspondence: akjaer@sund.ku.dk (A.K.); matthias.herth@sund.ku.dk (M.H.)
† Patricia E. Edem and Jesper T. Jørgensen contributed equally to this work.

Academic editor: Anne Roivainen and Xiang-Guo Li

Chromatographic Analysis

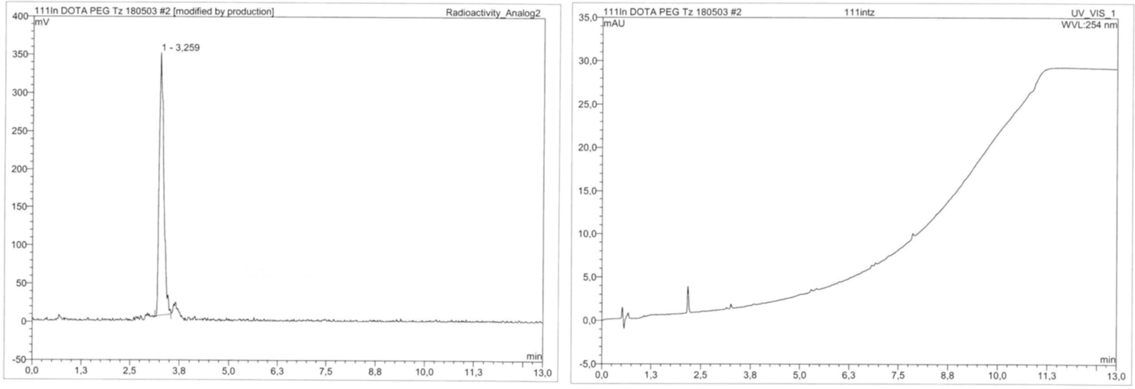

Figure S1. HPLC chromatograms of [^{111}In]**2** (R_t = 3.3 min). Radiochromatogram (left) and UV-visible (λ = 254 nm) HPLC chromatogram (right).

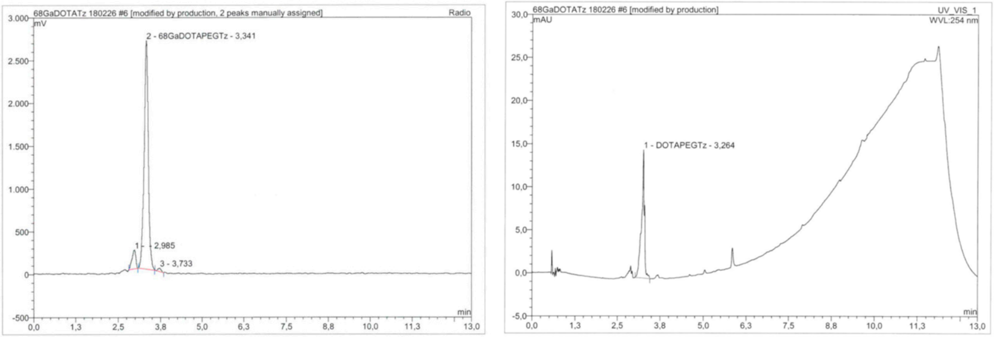

Figure S2. HPLC chromatograms depicting [^{68}Ga]**3** (R_t = 3.3 min) in the radiochromatogram (left) and **1** (R_t = 3.3 min) in the UV-visible (λ = 254 nm) HPLC chromatogram (right).

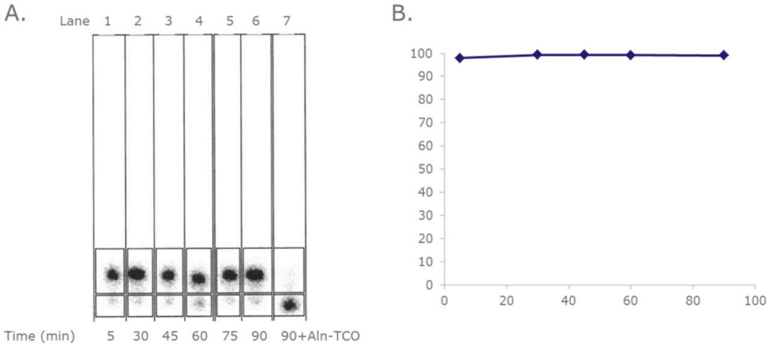

Figure S3. RP-radioTLC of [^{68}Ga]**3** after incubation in 10% EtOH/PBS for 5, 30, 45, 60, 75 and 90 min (lanes 1–6) and after incubation in 10% EtOH/PBS for 90 min followed by the addition of **4** in saline (lane 7) (**A**). In vitro stability of in 10% EtOH/PBS (**B**).

Pretargeted bone imaging

	**Control [^{111}In]2 (%ID/g)**		**Control [^{68}Ga]3 (%ID/g)**
	**SPECT 2 h**	**SPECT 22 h**	**PET 2 h**
**Shoulder**	0.06 ± 0.03	0.07 ± 0.03	0.13 ± 0.02
**Knee**	0.06 ± 0.03	0.07 ± 0.03	0.4 ± 0.2
**Muscle**	0.2 ± 0.1	0.04 ± 0.02	0.06 ± 0.02
**Heart**	0.12 ± 0.01	0.04 ± 0.01	0.3 ± 0.1

Table S1. Uptake values in selected tissues from SPECT scans 2 h and 22 h after injection of [^{111}In]**2** in untreated BALB/c mice (n = 3) and from PET scans 2 h after injection of [^{68}Ga]**3** in untreated BALB/c mice (n = 4). Data is given as mean ± standard error of mean (SEM).

Pretargeted tumour imaging

	**Control [^{111}In]2 (%ID/g)**		**Control [^{68}Ga]3 (%ID/g)**
	**SPECT 2 h**	**SPECT 22 h**	**PET 2 h**
**Tumour**	0.4 ± 0.1	0.08 ± 0.01	0.19 ± 0.03
**Heart**	0.2 ± 0.5	0.02 ± 0.03	0.18 ± 0.03
**Muscle**	0.5 ± 0.3	0.04 ± 0.02	0.17 ± 0.03

Table S2. Uptake values from [^{111}In]**2** SPECT/CT (n = 3) and [^{68}Ga]**3** PET/CT (n = 4) in selected tissues in nude BALB/c mice bearing subcutaneous LS174T tumour xenografts without pre-treatment with **5**. Data is given as mean ± standard error of mean (SEM).
